# Inactivation Effect of Violet and Blue Light on ESKAPE Pathogens and Closely Related Non-pathogenic Bacterial Species – A Promising Tool Against Antibiotic-Sensitive and Antibiotic-Resistant Microorganisms

**DOI:** 10.3389/fmicb.2020.612367

**Published:** 2021-01-13

**Authors:** Katharina Hoenes, Richard Bauer, Tobias Meurle, Barbara Spellerberg, Martin Hessling

**Affiliations:** ^1^Institute of Medical Engineering and Mechatronics, Ulm University of Applied Sciences, Ulm, Germany; ^2^Institute of Medical Microbiology and Hygiene, University Hospital Ulm, Ulm, Germany

**Keywords:** visible light irradiation, photoinactivation, antimicrobial blue light, ESKAPE pathogens, non-pathogen, 405 nm, 450 nm

## Abstract

Due to the globally observed increase in antibiotic resistance of bacterial pathogens and the simultaneous decline in new antibiotic developments, the need for alternative inactivation approaches is growing. This is especially true for the treatment of infections with the problematic ESKAPE pathogens, which include *Enterococcus faecium, Staphylococcus aureus, Klebsiella pneumoniae, Acinetobacter baumannii, Pseudomonas aeruginosa*, and *Enterobacter* species, and often exhibit multiple antibiotic resistances. Irradiation with visible light from the violet and blue spectral range is an inactivation approach that does not require any additional supplements. Multiple bacterial and fungal species were demonstrated to be sensitive to this disinfection technique. In the present study, pathogenic ESKAPE organisms and non-pathogenic relatives are irradiated with visible blue and violet light with wavelengths of 450 and 405 nm, respectively. The irradiation experiments are performed at 37°C to test a potential application for medical treatment. For all investigated microorganisms and both wavelengths, a decrease in colony forming units is observed with increasing irradiation dose, although there are differences between the examined bacterial species. A pronounced difference can be observed between Acinetobacter, which prove to be particularly light sensitive, and enterococci, which need higher irradiation doses for inactivation. Differences between pathogenic and non-pathogenic bacteria of one genus are comparatively small, with the tendency of non-pathogenic representatives being less susceptible. Visible light irradiation is therefore a promising approach to inactivate ESKAPE pathogens with future fields of application in prevention and therapy.

## Graphical Abstract

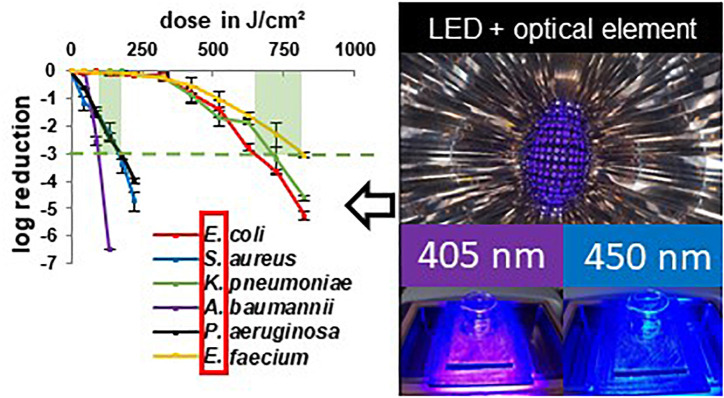

## Introduction

Hospital acquired infections (HAIs) challenge the health care sector (World Health Organization, 2017) and affect millions of patients each year. The increasing development of antibiotic resistances is worsening the situation creating an urgent need for establishing new anti-infective strategies. Consequently, to expedite the utilization of new technologies is critical, as the increasing prevalence of multidrug resistant microorganisms in hospitals became a serious threat for public health (Boucher et al., 2009). Most of the ESKAPE pathogens appear on the World Health Organization (WHO) list of the most problematic microbial species resulting in an appeal to concentrate research efforts on this topic. For 2007, the European Centre for Disease and Control (ECDC) estimated the death of 25,000 patients in the European Union, Norway and Iceland and an additional 900 million Euros hospital costs due to five selected antibiotic-resistant species (European center for disease prevention and control, 2009), completely intersecting with the ESKAPE pathogens. In 2016, HAI developed in 8.4% of patients staying in an intensive care unit for more than 2 days (European center for disease prevention and control, 2018).

Among research efforts to find alternative antimicrobial approaches, light-based technologies experience an intensified investigation (Mulani et al., 2019). Especially irradiation treatment with wavelengths in the range of visible light is often indicated as a possible antibiotic alternative (Wang et al., 2017; Hamblin and Abrahamse, 2019). Indeed, there seem to be multiple advantages. Due to current progress in the development of light emitting diodes (LED) this method is easily available, durable, sustainable and cost-effective. Several implementations in hospital-associated applications have been suggested. The equipment of endotracheal tubes with blue LEDs might intervene, where other antimicrobial approaches are struggling for many years to prevent ventilator associated pneumonia (VAP; Sicks et al., 2020). Endoscopically delivered light reduced gastric *Helicobacter pylori* infection (Ganz et al., 2005), urinary tract infection could successfully be treated in a rat model (Huang et al., 2018) and biofilm growth on catheter material was demonstrated to be reduced by the influence of irradiation (Vollmerhausen et al., 2017). The development of a light diffusing fiber delivery system (Shehatou et al., 2019), a minimally invasive ingestible capsule (Romano et al., 2016), the investigation of a continuous surface disinfection of hospital rooms during the patient’s presence (Maclean et al., 2013) and a whole range of different patents (Ahmed et al., 2018), demonstrate simultaneously the emerging significance of light-based technologies and their applicability.

The mechanisms for visible light inactivation is based on endogenous photosensitizers present in bacterial cells, which absorb photons of a certain wavelength converting the photosensitizers into an excited state. Those can interact with ambient oxygen to form reactive oxygen species (ROS) (Hamblin and Abrahamse, 2020), which cause damage to several intracellular targets (Dai et al., 2013; Adair and Drum, 2016; Kim and Yuk, 2017; Djouiai et al., 2018; Chu et al., 2019; Hyun and Lee, 2020). The violet wavelength of 405 nm was proven to be especially effective (Maclean et al., 2008; Endarko et al., 2012) tracing back to endogenous porphyrins, which are recognized as responsible photosensitizers (Ashkenazi et al., 2003; Hamblin et al., 2005; Lipovsky et al., 2009). The blue wavelength of 450 nm also has a considerable inactivation impact, albeit weaker than for 405 nm (Hessling et al., 2017; Plavskii et al., 2018; Tomb et al., 2018) but may be preferable for clinical applications due to higher tissue penetration depth and lower absorption by blood. For 450 nm flavins are considered as responsible photosensitizer (Cieplik et al., 2014; Plavskii et al., 2018; Hoenes et al., 2019).

It has been noticed that differences exist in the way, in which microbial genera react to the exposure to visible light of the same wavelength (Hessling et al., 2017; Tomb et al., 2018). However, the underlying causes are not totally resolved. Varying compositions and concentrations of endogenous photosensitizers might play a role, as those are the responsible mediators between light and bacterial damage. Due to differing investigation methods, the comparison of test results for different strains is problematic. It is hard to determine if variables arise from biological factors or the different treatment and environmental properties. Parameters like pH of the culture medium (Kjeldstad et al., 1984), specific culture conditions and growth phase (Keshishyan et al., 2015; Abana et al., 2017; Biener et al., 2017; Fyrestam et al., 2017) as well as pH, salt concentration and temperature during irradiation (Ghate et al., 2015; Keshishyan et al., 2015; McKenzie et al., 2016), have been demonstrated to influence bacterial inactivation. Barneck et al. (2016) found that the irradiation effect is dose-dependent and that different irradiation intensities did not play a role concerning the inactivation result at a specific dose, independent of the irradiation time. Nevertheless, this was only a single study, so it might be risky to rely on the dose-dependency when comparability is desired.

Our aim was therefore to perform all our investigations on pathogenic and non-pathogenic representatives of different genera with the same test protocol. In this study, we wanted to examine whether pathogenic bacterial species and closely related non-pathogenic species respond in the same manner to visible light irradiation at wavelengths of 405 and 450 nm. Therefore, a pathogenic strain and a non-pathogenic relative were investigated in comparison. To come up with new methods capable to cope with ESKAPE pathogens we chose those 6 species for our experiments. In total, we thus investigated 11 strains, as no non-pathogenic *Klebsiella* strain is available.

## Materials and Methods

### Bacterial Strains and Cultivation

*Enterococcus moraviensis* (DSM 15919), *Staphylococcus carnosus* (DSM 20501), *Acinetobacter kookii* (DSM 29071), *Pseudomonas stutzeri* (DSM 5190), *Escherichia coli* (DSM 1607), and *Enterococcus faecium* (DSM 17050) were obtained from DSMZ (Deutsche Sammlung für Mikroorganismen und Zellkulturen, Braunschweig, Germany). *Staphylococcus aureus* (ATCC 43300), *Klebsiella pneumoniae* (ATCC 700603), *Acinetobacter baumannii* (ATCC 19606), and *Pseudomonas aeruginosa* (ATCC 27853) were obtained from American Type Culture Collection (ATCC, Manassas, VA, United States). *Escherichia coli* BSU1286 was obtained from the Institute of Medical Microbiology and Hygiene (University Hospital Ulm, Germany). All strains were cultivated to mid-exponential phase and then centrifuged at 7,000 *g* for 5 min. The resultant pellet was resuspended in phosphate buffered saline (PBS) and washed in PBS, before the suspension was diluted to the desired population density between 5 × 10^7^ and 10^8^ colony forming units per ml (CFU/ml) for experimental use. All experiments were conducted in PBS to avoid the possible photosensitizing impact and absorbance of medium ingredients. Media that were used for cultivation are listed in Table 1.

**TABLE 1:** Overview of the strains investigated in this study in this study and their specific media.

strain	Pathogenicity	Resistances	Medium reference	Ingredients per 1000 ml
*E. moraviensis*, *S. carnosus*	-	–	M92	30 g tryptic soy broth (Sigma-Aldrich Chemie GmbH, München, Germany), 3 g yeast extract (Merck KGaA, Darmstadt, Germany)
*A. kookii*	-	–	M220	15 g peptone from casein (VWR international, Leuven Belgium), 5 g peptone from soymeal (Sigma-Aldrich Chemie GmbH, München, Germany), 5 g sodium chloride (VWR international, Leuven Belgium)
*P. stutzeri*	-	–	M1	5 g peptone from casein (VWR international, Leuven Belgium), 3 g meat extract (VWR international, Leuven Belgium)
*E. coli* HB101 K12	-	–	LB	10 g tryptone (VWR international, Leuven, Belgium), 5 g yeast extract (Merck KgaA, Darmstadt, Germany), 10 g sodium chloride (VWR international, Leuven Belgium)
*E. faecium*	+	VRE	THY	36.4 g Todd–Hewitt Broth (Oxoid, Basingstoke, United Kingdom), 5 g yeast extract (BD, Sparks, MD, United States)
*S. aureus*	+	MRSA	THY	36.4 g Todd–Hewitt Broth (Oxoid, Basingstoke, United Kingdom), 5 g yeast extract (BD, Sparks, MD, United States)
*K. pneumoniae*	+	ESBL	THY	36.4 g Todd–Hewitt Broth (Oxoid, Basingstoke, United Kingdom), 5 g yeast extract (BD, Sparks, MD, United States)
*A. baumannii*	+	–	LB	10 g tryptone (Gibco, Detroit, MI), 5 g yeast extract (BD, Sparks, MD, United States), 10 g sodium chloride (AppliChem, Darmstadt, Germany)
*P. aeruginosa*	+	–	THY	36.4 g Todd–Hewitt Broth (Oxoid, Basingstoke, United Kingdom), 5 g yeast extract (BD, Sparks, MD, United States)
*E. coli*	+	ESBL	LB	10 g tryptone (Gibco, Detroit, MI), 5 g yeast extract (BD, Sparks, MD, United States), 10 g sodium chloride (AppliChem, Darmstadt, Germany)

*ESBL, extended spectrum beta-lactamases; MRSA, methicillin resistant *S. aureus*; VRE, vancomycin resistant enterococcus. The declaration of biosafety level served as indicator for the pathogenicity.*

### Irradiation Setup

**FIGURE 1:**
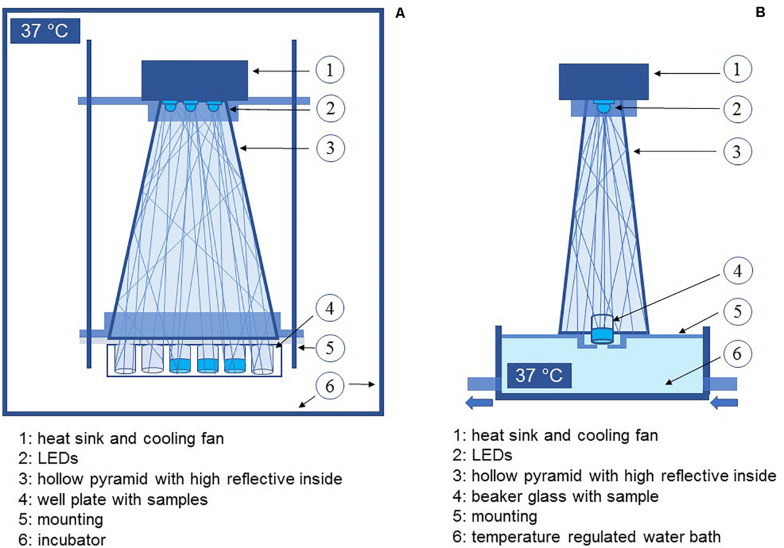
Schematic depiction of the experimental setup for experiments with pathogenic bacteria **(A)** and non-pathogenic bacteria **(B)**.

Two different wavelengths were applied for irradiation. Due to safety regulations the strains were investigated in two groups and in two laboratories with test setups slightly adapted to laboratory conditions, but with the same parameters. A truncated hollow pyramid with a high reflective inside, already described before in Hoenes et al., 2019, ensured that the sample area was irradiated homogenously. LEDs were mounted to a heat sink, which was actively cooled with a fan during experiments to avoid heating the sample (Figure 1). Experiments were performed in separate vessels with 11 mm diameter. For irradiation at the violet wavelength, LEDs with a peak wavelength of 405 nm were applied. The second wavelength was chosen from the blue wavelength range at 450 nm. For both wavelengths, an intensity of 20 mW/cm^2^ was selected. Irradiation intensity was measured by means of an optical power meter OPM150 (Qioptiq, Göttingen, Germany).

As we wanted to refer to therapeutic applications taking place at or in the human body the experiments were conducted at 37°C. For pathogenic microorganisms, the whole setup was placed inside an incubator. Samples of non-pathogenic strains were heated by a temperature regulated water bath ICC basic (IKA, Staufen, Germany), with tempered water passing the lower third of the vessel continuously. The sample temperature was checked at each sampling interval with an infrared thermometer (Raytek Fluke Process Instruments GmbH, Berlin, Germany).

Samples were drawn in certain time intervals with increasing doses and plated on nutrient agar. The media used for agar plates have been the same as the cultivation media for non-pathogenic and sheep blood agar plates (TSA + SB, Oxoid, Basingstoke, United Kingdom) for pathogenic strains. After incubation at 37°C for 24–48 h grown colonies were enumerated manually. The resultant count was converted to CFU/ml and expressed as log reduction in comparison to the starting concentration. Each experiment was performed in triplicates and repeated at least three times.

## Results

**FIGURE 2:**
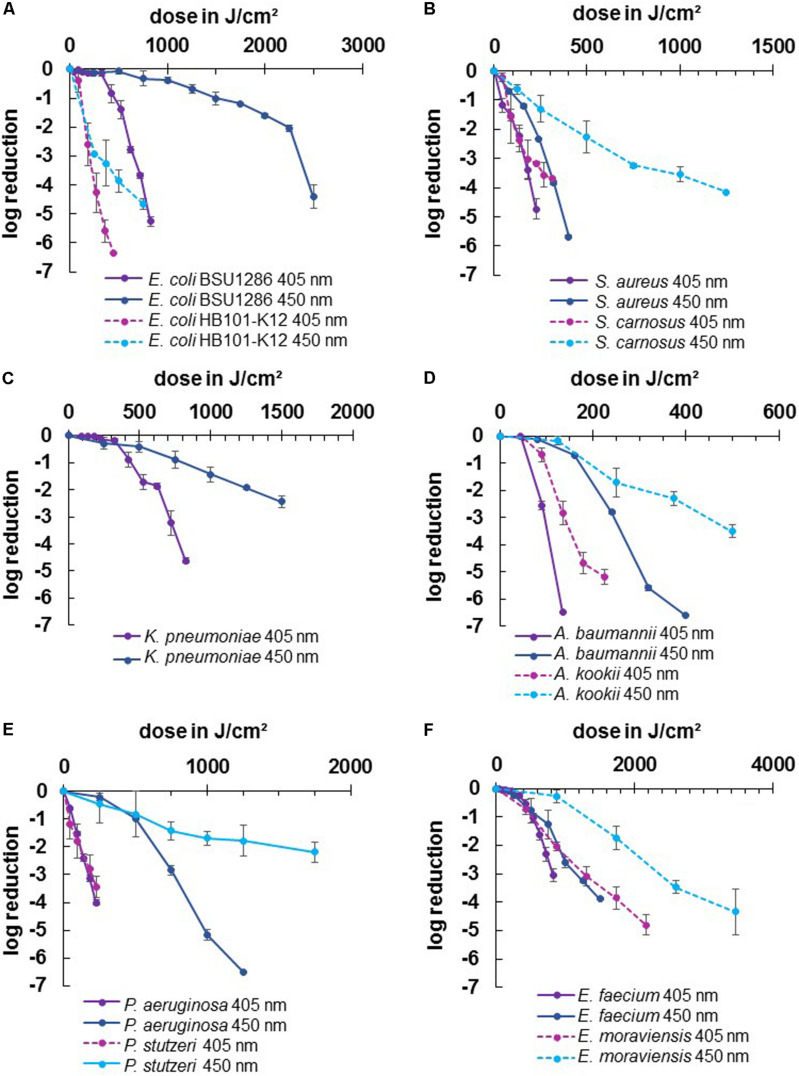
Inactivation results for pathogenic ESKAPE-strains and non-pathogenic relatives for 405 and 450 nm: *E. coli* BSU1286/*E. coli* HB101 K12 **(A)**, *S. aureus*/*S. carnosus* **(B)**, *K. pneumoniae* **(C)**, *A. baumannii*/*A. kookii* **(D)**, *P. aeruginosa*/*P. stutzeri* **(E)**, *E. faecium*/*E. moraviensis* **(F)**. Error bars represent standard deviation of three independent experiments.

In this study we wanted to evaluate the irradiation susceptibility of different microbial genera and potential differences between closely related pathogenic and non-pathogenic bacterial species. In Figure 2 the inactivation results for the wavelengths 405 and 450 nm are depicted for two different species in each illustration - one pathogenic and one non-pathogenic of a certain genus, to compare whether there are differences in their inactivation profile. The dotted lines portray the non-pathogenic representative, while the solid lines represent the pathogenic species. There was no considerable decrease in most non-irradiated controls. However, the *E. moraviensis* control was reduced by 1.38 log at the end of the longer 450 nm experiment at 48 h. Therefore, the log decrease in the control at this strain was subtracted from the irradiation value at each sampling point to demonstrate the sole light impact. Likewise, *A. kookii* results were corrected in this manner. For all other strains, the decrease in the control was so low to not perceptibly affect the irradiation results.

For all tested strains, the violet wavelength of 405 nm exhibited a stronger inactivation effect compared to the blue wavelength of 450 nm (Figure 2). However, comparing the two wavelengths between pathogenic and non-pathogenic species, there were two cases in which 450 nm on the pathogenic strain was as effective as 405 nm on the non-pathogenic strain at a similar dose. For *S. aureus* we achieved 3.84 log reduction at 320 J/cm^2^ with 450 nm while *S. carnosus* required 315 J/cm^2^ of 405 nm to be inactivated by 3.70 log (Figure 2B). Likewise, for *E. faecium* 3.26 log at 1250 J/cm^2^ was achieved with 450 nm, while *E. moraviensis* was reduced by 3.08 log when exposed to 1296 J/cm^2^ of 405 nm (Figure 2F).

All strains exhibited the typical behavior for visible light inactivation with a non-monoexponential shape, showing a linear relationship of inactivation and dose in a semi-logarithmic representation. In some cases, a so called “shoulder” occured at lower doses indicating the ability of bacteria to overcome small damages. This slow initial reaction particularly became apparent for 450 nm irradiation of *E. coli* BSU1286, showing a slow decrease up to a dose of 2250 J/cm^2^, followed by a steeper slope (Figure 2A). In addition, at 450 nm *P. aeruginosa* illustrated a distinct development of this behavior (Figure 2E).

Both applied wavelengths were successful at inactivating the strains investigated here. However, the doses necessary to achieve a certain log reduction were varying, especially between different genera. The highest dose of 450 nm irradiation for a reduction of 3 or more log levels, had to be applied to *E. coli* BSU1286, with 2500 J/cm^2^ for 4.4 log reduction (Figure 2A). Meanwhile, only 320 J/cm^2^ of 450 nm had to be applied to *A. baumannii* for a 5.60 log reduction. Concerning 405 nm Acinetobacter likewise appeared to be the most susceptible genus with only 225 J/cm^2^ exposure that was necessary to reduce the non-pathogenic representative *A. kookii* by 5.19 log (Figure 2D). The microorganisms most difficult to diminish with 405 nm have been enterococci with *E. faecium* being inactivated by 3.06 log at 825 J/cm^2^ and *E. moraviensis* requiring 1296 J/cm^2^ for 3.08 log reduction (Figure 2F).

The difference in the effectiveness of the two wavelengths compared has been most apparent for pseudomonads (Figure 2E). For *P. aeruginosa* in addition the extensive development of a shoulder at 450 nm was observable, whereas the 405 nm inactivation progress illustrated an almost straight line, representing an exponential decline. Furthermore, the pathogen *E. coli* representative showed huge susceptibility differences between the two wavelengths, which is essentially caused by the low inactivation efficiency at 450 nm in this case (Figure 2A).

For 405 nm the non-pathogenic and pathogenic representatives of the same species exhibited similar behavior. Non-pathogenic strains tended to be rather less susceptible to 405 nm irradiation, becoming apparent when directly opposing log reductions. Indicating the non-pathogenic strain following by the pathogenic, reductions of 3.16/4.74 log at 225 J/cm^2^ for staphylococci, 0.69/2.55 log at 90 J/cm^2^ for Acinetobacter, 2.06/3.05 at 864/825 J/cm^2^ for enterococci, and 3.43/4.01 log at 225 J/cm^2^ for pseudomonads respectively, have been achieved. Only for *E. coli* the proportions are inverted with 6.36/0.84 log at 450/425 J/cm^2^, with the disparity being especially vast here. For the pathogenic *E. coli* strain BSU1286 a dose of 825 J/cm^2^ was necessary to reach a reduction of 5.26 log, which means that an approximately doubled dose was needed for the same reduction compared to the non-pathogenic relative (Figure 2A).

The same trend as for 405 nm became apparent for 450 nm. Likewise, the non-pathogenic strain was less susceptible than its pathogenic relative. Here we achieved log reductions of 2.27/5.69 at 500/400 J/cm^2^ for staphylococci, 2.30/6.61 at 375/400 J/cm^2^ for Acinetobacter, 1.73/3.89 log at 1728/1500 J/cm^2^ for enterococci, and 1.78/5.17 log at 1250 J/cm^2^ for pseudomonads, again first naming the non-pathogenic followed by the pathogenic strain. Again, the pathogenic *E. coli* strain was in contrary much less susceptible than the non-pathogenic strain with 4.66/0.33 log reduction at 750 J/cm^2^ of 450 nm. To reach an inactivation of 4.41 log the suspension had to be exposed to 2500 J/cm^2^ of 450 nm (Figure 2A).

Altogether, the least differences between pathogenic and non-pathogenic relative occurred for pseudomonads referring to the wavelength of 405 nm (Figure 2E). However, concerning 450 nm irradiation *Pseudomonas* together with *Escherichia* are the genera at which the differences between pathogenic and non-pathogenic strains investigated became most evident, albeit in opposite direction. Contemplating the general strain differences across all microorganisms, the differences at 450 nm irradiation had been more evident than for 405 nm.

**FIGURE 3:**
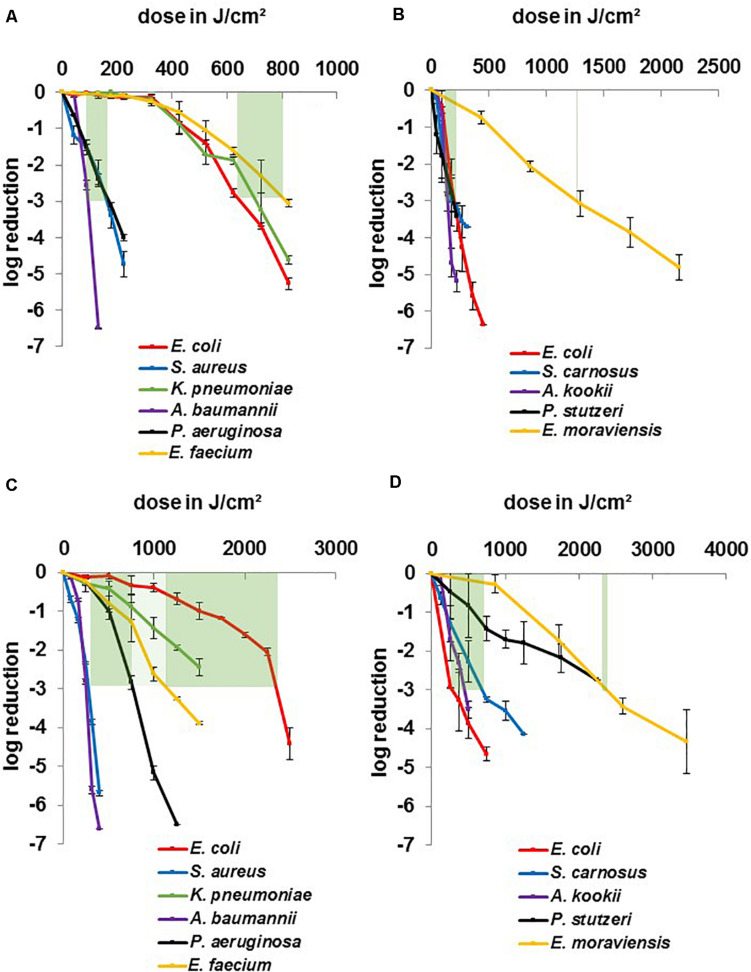
Inactivation results for pathogenic ESKAPE-strains and non-pathogenic relatives for 405 and 450 nm grouped by wavelength: pathogenic strains at 405 nm **(A)**, non-pathogenic strains at405 nm **(B)**, pathogenic strains at 450 nm **(C)**, non-pathogenic strains at 450 nm **(D)**. Green bars mark a dose range for similar behaving microorganisms for a 3 log reduction. Error bars represent standard deviation of three independent experiments.

In Figure 3 where data have been categorized concerning better comparability of different genera, it becomes apparent that 3 strains especially stand out regarding their insusceptibility. *K. pneumoniae*, *E. coli* BSU1286, and *E. faecium* (Figure 3A) provide a strong contrast compared to the other pathogen representatives at 405 nm and clearly divide the microorganisms in two different susceptibility groups. To achieve a 3 log reduction, the dose ranges of 100–175 J/cm^2^ compared to 650–800 J/cm^2^ had to be applied, respectively. Non-pathogenic strains (Figure 3B) lie closer together with a range of 125–200 J/cm^2^ with only *E. moraviensis* as outlier with around 1200 J/cm^2^ of 405 nm. Yet it is necessary to mention here, that a non-pathogenic *Klebsiella* strain was not available for testing.

A similar distribution is observable for the non-pathogenic strains at 450 nm (Figure 3D) with a dose range of 350–750 J/cm^2^ and *E. moraviensis* as well as *P. stutzeri* at around 2400 J/cm^2^, though it has to be noted that the field had widened at this wavelength. While *E. coli* and *E. moraviensis* required a doubled dose comparing 405 to 450 nm, *S. carnosus* and *A. kookii* exhibited even more reduced susceptibility at 450 nm, while still lying in the range of 2 to 5-fold dose increase, which is found in the literature comparing 405 and 450 nm (Hessling et al., 2017; Tomb et al., 2018). *P. stutzeri* required even the 12-fold dose at 450 nm compared to 405 nm, which is an unusual finding regarding the literature and was likewise not observed for the pathogenic *P. aeruginosa*.

The 450 nm dose range of the more susceptible pathogenic group (Figure 3C) was 250–750 J/cm^2^, indicating a 2 to 5-fold increase at the blue wavelength. Similarly, as for the non-pathogenic strains, it is observable that *P. aeruginosa* especially required higher doses at 450 nm, as mentioned before. *E. faecium* had not pulled apart extremely, but *K. pneumoniae* and *E. coli* BSU1286 spread the field to a dose range of 1,200–2,400 J/cm^2^. Overall, for pathogenic strains at 450 nm the dose distribution for different genera is evenly distributed. While the susceptibility tendencies of different genera are still visible, no similar clear separable categories as for 405 nm could be drawn.

## Discussion

Photoinactivation with visible light has recently received increased interest due to the demand for the development of new disinfection technologies. Multiple studies investigating single bacterial species with a certain test setup at a specific wavelength have been published. However, extensive differences were observed in these data collections probably caused by a lack of uniform methodologies (Hessling et al., 2017; Tomb et al., 2018). Only few studies compare multiple strains with the same test protocol. Therefore, we aimed to investigate all ESKAPE pathogens under comparable conditions, including the comparison of related pathogenic and non-pathogenic species. As literature data about non-pathogenic microorganisms are still scarce we mainly concentrated on the pathogenic representatives for a comparison with the literature data to evaluate the achieved results.

Maclean et al. (2009) investigated 405 nm irradiation at 10 mW/cm^2^ against a broad range of microorganisms including all ESKAPE-pathogens. Their findings concerning the sensitivity of different genera is comparable to the results obtained here. *A. baumannii*, *S. aureus*, and *P. aeruginosa* are more susceptible to visible light, while for *E. coli*, enterococci and *K. pneumoniae* higher doses are necessary for 1 log reduction (Figure 2). Compared to our results there is a factor of approximately 2 in the dose for 1 log reduction of *E. coli*, enterococci and *K. pneumoniae* compared to Maclean et al. (2009), but the tendencies are similar, including the necessity of an exposure of about 300 J/cm^2^ for enterococci until inactivation progress is noticeable. In addition, the inactivation dose of 42.9 J/cm^2^ for 1 log reduction of *P. aeruginosa* is comparable to the 57.1 J/cm^2^ result achieved here.

Gupta et al. (2015) investigated clinical isolates from infectious hip and knee arthroplasties including all ESKAPE species except *A. baumannii* at 123 mW/cm^2^ of 405 nm irradiation. The study indicated the dose needed to reach an approximate 5 log reduction, which was their measured maximum. With 441.9 J/cm^2^ for a calculated 1 log average inactivation their results fit well to the calculated 492.2 J/cm^2^ for 1 log *E. coli* BSU1286 inactivation achieved here. For *K. pneumoniae* and *S. aureus* the averaged 1 log exposures were lower than in this study, even though clinical isolates were used by Gupta et al. (2015), which are often considered to be more challenging to inactivate. For *E. faecium* Halstead et al. (2016) delivered a value of 393 J/cm^2^ for 1 log reduction for a clinical isolate at 400 nm irradiation, while the strain investigated here required 525 J/cm^2^.

Concluding, the results for the necessary dose at 405 nm irradiation in this study are relatively high, especially concerning the inactivation of Enterobacteriaceae - including *Klebsiella* and *Escherichia* – and enterococci. For *S. aureus* there are several studies coming to similar conclusions with doses between 50.2 and 61.6 for a 1 log reduction (Enwemeka et al., 2008; Guffey et al., 2013; McKenzie et al., 2016). Furthermore, our data is in agreement with the literature that *Acinetobacter baumannii*, declared as one of the most problematic species in the WHO priority list (World Health Organization, 2017), is the most susceptible ESKAPE pathogen at 405 nm irradiation.

Irradiation around 450 nm has not been tested as extensively as 405 nm, hence the literature data available to compare with the results obtained here are limited. As expected, the doses required for inactivation have been higher at 450 nm for all strains investigated compared to 405 nm. Interestingly, a linear fit applied to the 450 nm data of *E. faecium* and *K. pneumoniae* delivered coefficients of 0.9453 and 0.9534, respectively, while “shoulders” existed for 405 nm irradiation. In contrast, for *S. aureus* and *P. aeruginosa* a linear fit was suitable at 405 nm, whereas a distinct “shoulder” appeared at 450 nm treatment. It was noticed before (Schmid et al., 2019) that there seems to be a correlation between the length of the wavelength, the duration of the slow initial inactivation and the occurrence of a shoulder. Webb and Brown (1976) already described similar observations concerning the wavelength dependency of the shoulder-effect in 1976 for *E. coli*.

For *P. aeruginosa* two very diverse literature results for 450 nm irradiation exist. Tomb et al. (2018) reported 91.9 J/cm^2^ (Keshishyan et al., 2015) and 428.6 J/cm^2^ (Decarli et al., 2016) for a 1 log reduction at 450 and 460 nm, respectively. We measured 0.98 log at 500 J/cm^2^ and can therefore reproduce the value from the second study, although the first study was conducted at 37°C and the second at room temperature. Even though longer wavelengths were considered to have less impact on bacteria, the wavelength of 460 nm is probably not very different from 450 nm, as the flavin absorption peak is relatively broad. For *S. carnosus* we previously determined an increase of 12% for the required dose at 460 nm compared to 450 nm when conducting an action spectrum (Hoenes et al., 2019).

Lui et al. (2016) investigated *E. faecalis* at 455 nm irradiation for drinking water disinfection and found a required exposure dose of 410 J/cm^2^ for a 1 log reduction. With 0.78 log at 500 J/cm^2^ our results for *E. faecium* are comparable.

Some studies were conducted on pathogenic *E. coli* at wavelengths between 450 and 455 nm (Lipovsky et al., 2010; Keshishyan et al., 2015; Lui et al., 2016) ranging from approximately 100–300 J/cm^2^ for 1 log inactivation but none of them is close to our results with 1500 J/cm^2^ for 1.00 log. Within this study the *E. coli* strain stands out, although the majority of the data for all strains investigated here already rank at the upper end of visible light inactivation data.

The investigated *S. aureus* strain however was inactivated by 1.22 log at 160 J/cm^2^ of 450 nm, while literature data suggest a 1 log dose of around 300 J/cm^2^ at 450 nm at 37°C (Keshishyan et al., 2015), 400 J/cm^2^ at 455 nm (Lipovsky et al., 2010), or 300 J/cm^2^ at 460 nm (Decarli et al., 2016) for methicillin-sensitive strains.

To our knowledge, there are no data available for the inactivation of any *Acinetobacter* or *Klebsiella* strain with 450 nm light.

However, it is difficult to compare the data on bacterial inactivation of different research groups. Due to the lack of uniform test setups and protocols influences might occur that are not apparent at first sight. In this study, special attention was paid to a homogenous irradiation intensity by applying a reflective pyramid structure. Depending on the light source arrangement, without a homogenizing technique, great variations in intensity can occur due to the punctiform emission of LEDs, making it difficult to relate the achieved bacterial impact to a certain dose applied over time. A specificity for the results of this study is the investigation at 37°C, directing at achieving a benchmark for applications at human body temperature. As most literature data were obtained at room temperature (with varying degree of monitoring and regulation), this can make a direct comparison difficult. The research groups that obtained data at different temperatures tend to suggest that lower temperatures are favorable for visible light inactivation (McKenzie et al., 2014; Keshishyan et al., 2015; Kumar et al., 2015). This might partly explain the ranking of our data at the upper end compared to available reference values at room temperature.

This study is part of an ongoing development of an enhanced endotracheal tube. A potential protoype was presented earlier (Sicks et al., 2020). Utilizing the antimicrobial effects of photoinactivation and the applicability without adding external photosensitizers we aim to equip endotracheal tubes with a light source for prevention of VAP. The susceptibility investigation of ESKAPE microorganisms at the body core temperature of 37°C forms the foundation for further experiments under more realistic conditions. Data of this universal scientific state are nevertheless important and can likewise be used for multiple further medical developments. Catheter based approaches were already suggested (Vollmerhausen et al., 2017; Huang et al., 2018) as well as an ingestible capsule (Romano et al., 2016) against gastric ulcer, but also (burn) wound treatment is conceivable (Dai et al., 2013; Halstead et al., 2016).

It has been discussed before whether the Gram properties of microorganisms affect their susceptibility towards visible light (Maclean et al., 2009). We could not detect any tendency, as staphylococci and enterococci, the two Gram positive species investigated, exhibited very different inactivation behavior and belonged to different groups of susceptibility for both 405 and 450 nm (Figure 3). Confirmative, there was no distinction found between the entirety of Gram positive and Gram negative species by Tomb et al. (2018) when reviewing literature data, with both groups requiring around 200 J/cm^2^ of 405 nm irradiation in average for a 1 log reduction. The same absence of a clear difference between Gram properties was noticed in Hessling et al. (2017).

Except from the *E. coli* strains the irradiation at both wavelengths indicated a lower susceptibility for the non-pathogenic representatives investigated. Abana et al. (2017) investigated various *E. coli* strains, including a non-pathogenic strain, on their 455 nm light inactivation behavior. Comparable to our results the non-pathogenic strain was the most susceptible with approximately 2 log reduction at 120 J/cm^2^. Lacombe et al. (2016) however did not detect differences in response to 405 nm between pathogenic and non-pathogenic *E. coli* strains. Nevertheless, the majority of non-pathogenic strains have been less susceptible in our study. In the supplementary material of the review of Tomb et al. (2018) we found a reference to a *P. stutzeri* investigation, achieving a 1 log reduction at 60 J/cm^2^ of 405 nm. Our result with 3.43 log reduction at 225 J/cm^2^ of 405 nm can be converted to 67.4 J/cm^2^ necessary for 1 log reduction by a linear fit at log scale, which matches perfectly.

Virulence factors have been a recent topic of investigation within light-based approaches, as it was found that they will be destroyed by antimicrobial photodynamic inactivation (aPDI) using external photosensitizers for irradiation treatment (Kömerik et al., 2000; Tubby et al., 2009; Kato et al., 2013; Pourhajibagher et al., 2018). Bartolomeu et al. (2016) even found in their study investigating 6 *S. aureus* strains with varying properties, that those producing endotoxins were more susceptible to the aPDI treatment. Tang et al. (2007) noticed a stronger inactivation by irradiation for a MRSA strain compared with the corresponding ATCC strain, ascribed to the altered penicillin binding protein, which lead to a more permeable cell wall. A ROS induced impairment of factors for antimicrobial resistance or virulence, not only integral for the pathogenic properties but also fulfilling physiological performance in the microorganisms (Peterson, 1996), might therefore contribute to an additional reduction in viability. However, there might exist differences comparing aPDI and visible light irradiation, which is solely based on endogenous photosensitizers. In this study we therefore investigated whether similar tendencies are detectable for 405 and 450 nm irradiation without external dyes and if virulence plays a role for the behavior concerning irradiation.

The American Society for Microbiology (ASM) published a study, on important criteria for choosing appropriate surrogates for pathogens (Sinclair et al., 2012). For testing disinfection methods, it is recommended to choose a strain that is rather resistant compared to the target microorganisms, and thus testing a kind of worst case scenario. This criterion is fulfilled for all our non-pathogenic strains, besides *E. coli*.

The ASM further recommends to achieve a performance of 99.9% reduction in bactericidal tests (American Society for Microbiology, 2019). All investigated strains in this study reached this goal for irradiation with both 405 nm and 450 nm, besides *K. pneumoniae* and *P. stutzeri* at 450 nm for which the applied maximum dose of 1500 J/cm^2^ and 2250 J/cm^2^ led to a 2.44 log and a 2.75 log inactivation, respectively. The kinetics of the inactivation curves however suggest that 3 log can be reached at higher doses.

## Conclusion

To our best knowledge, this has been the first time that all ESKAPE pathogens have been investigated within one study and therefore with the same test methodology, not only on their susceptibility to 405 nm but also to 450 nm. All strains in this study could be inactivated significantly with both wavelengths.

It has also been the first time that related pathogenic and non-pathogenic bacterial species have been directly compared. In our investigation, non-pathogenic strains tend to be less susceptible than the pathogenic representatives at both wavelengths, with the differences being more pronounced at 450 nm. As non-pathogenic relatives are rather more difficult to inactivate, it is considered valid to choose non-pathogenic surrogates for the investigation of visible light photoinactivation.

An exception was noticed for *E. coli* BSU1286, where the pathogenic strain was far less susceptible, not only compared to the non-pathogenic relative, but also compared to all other strains and to the available literature data.

## Data Availability Statement

The raw data supporting the conclusions of this article will be made available by the authors, without undue reservation.

## Author Contributions

KH co-designed the study, analyzed the effect of 405 and 450 nm on non-pathogenic microorganisms, and wrote the manuscript. RB analyzed the effect of 405 and 450 nm on pathogenic microorganisms and edited the manuscript. TM constructed the irradiation setup, helped with the experiments on non-pathogenic microorganisms and edited the manuscript. BS and MH designed the study, supervised the study, and edited the manuscript. All authors contributed to the article and approved the submitted version.

## Conflict of Interest

The authors declare that the research was conducted in the absence of any commercial or financial relationships that could be construed as a potential conflict of interest.
